# Undetection of vector-borne viruses in equids of Galapagos Islands

**DOI:** 10.3389/fvets.2024.1411624

**Published:** 2024-06-07

**Authors:** Gina Zanella, Cécile Beck, José-Carlos Valle-Casuso, Madeline Anthony, Marilyn Cruz, Alberto Vélez, Rommel Lenin Vinueza, Gaëlle Gonzalez

**Affiliations:** ^1^Epidemiology Unit (EPIMIM), Laboratoire de Santé Animale, ANSES, Ecole Nationale Vétérinaire d’Alfort, Maisons-Alfort, France; ^2^ANSES, INRAE, Ecole Nationale Vétérinaire d'Alfort, UMR 1161 Virologie, Laboratoire de Santé Animale, Maisons-Alfort, France; ^3^ANSES, Laboratory for Animal Health in Normandy, Physiopathology and Epidemiology of Equine Diseases Unit, Goustranville, France; ^4^Mixed Technological Unit "Equine Health and Welfare - Organisation and Traceability of the Equine Industry" (UMT SABOT), Normandie, France; ^5^Agencia de Regulación y Control de la Bioseguridad y Cuarentena para Galápagos (ABG), Puerto Ayora, Ecuador; ^6^Escuela de Medicina Veterinaria, Universidad San Francisco de Quito, Quito, Ecuador

**Keywords:** WNV, USUV, EIAV, West Nile, Usutu, equine infectious anemia, Galapagos Islands, Ecuador

## Abstract

Domestic species, including equids, were introduced in the Galapagos Islands in the XIX century. Equine vector-borne diseases are circulating in South America but their occurrence in the Galapagos Island was unknown. The objective of this study was to detect the occurrence of West Nile virus (WNV), Usutu virus (USUV) and equine infectious anemia virus (EIAV) in the four Galapagos Islands raising equids if they were present at a prevalence >1%. Serum samples were collected from 411 equids belonging to 124 owners from April to July 2019. All the results were negative to the ELISA tests used suggesting that WNV, USUV and EIAV are not circulating in the equine population of the Galapagos Islands.

## Introduction

The Galapagos Islands are a set of insular territories 960 km off the coast of Ecuador and an Ecuadorian province. They have been listed as a UNESCO World Heritage Site. Only 3% of their territory is colonized while the remaining 97% belongs to the Galapagos National Park. The introduction of livestock into the islands dates back from the beginning of the XIX century. In 1832, there was a massive introduction of productive domestic species, including equids, which may have carried parasites or pathogens (1). In 2003, animal importation from mainland Ecuador was banned, except for day-old chicks. Equids are present among the four colonized islands: Santa Cruz, Isabela, San Cristóbal and Floreana. Equid movements are allowed between the Galapagos Islands for animal genetic improvement. Surveillance of viral equid disease through laboratory diagnosis had never been conducted. Considering the movement of animals between mainland Ecuador and the Galapagos Islands in the past, the likelihood of the occurrence of viruses affecting the equids into the islands could not be excluded. Vector-borne viruses could have also been introduced by vectors coming from mainland.

West Nile virus (WNV) and Usutu virus (USUV) are vector–borne viruses that can affect equids and humans (2). They belong to the family *Flaviviridae*, genus *Flavivirus*, and are responsible for multiple outbreaks of disease in different countries of the world. WNV and USUV transmission cycles include wild birds as amplifying hosts and ornithophilic mosquitoes as vectors but can also infect and cause disease in horses and humans, which serve as incidental dead-end hosts (3, 4). WNV is one of the pathogenic agents that can lead to equine neurological clinical signs, although the infection is not usually accompanied by presentation of clinical illness (5). WNV is endemic in parts of Africa, Europe, the Middle East, and Asia, and since 1999 has spread to North America, Mexico, South America, and the Caribbean (6). WNV has been detected in mainland Ecuador through serological surveys in equids (7) and also in Colombia, an Ecuadorian neighboring country (8). USUV is particularly pathogenic in a few species of birds and has been detected in many mammalian species, including equids, considered to be dead-end hosts (9). USUV has not been reported in Latin America but in view of the emergence of different arboviral diseases, such as chikungunya and Zika, it can be considered as a new emerging threat (10).

Equine infectious anemia (EIA) is caused by a lentivirus and can be transmitted mechanically by biting flies (the virus does not replicate in the vector) (11). EIA virus (EIAV) infection leads to recurring episodes of fever in equids, thrombocytopenia, and wasting symptoms. EIA has been detected in different regions in Brazil (12–14) and Argentina (15) and several outbreaks were reported in different regions in mainland Ecuador (16).

The present study aimed to detect the occurrence of WNV, USUV and EIAV in the Galapagos Islands where equids are raised.

## Method

### Study population

The Galapagos veterinary services had estimated that around 650 equids are present in the islands of Santa Cruz (~ 230 equids), Isabela (~ 270 equids), San Cristóbal (~ 100 equids) and Floreana (~ 30 equids). The equids often belong to farmers who raise other species, mainly cattle, and are used for farm work, transportation or leisure purposes. There are approximately 132 equid owners in the four islands (63 in Santa Cruz, 34 in Isabela, 26 in San Cristobal, 9 in Floreana) and between one and 15 equids per farm.

### Sampling

For each island, the sample size was calculated to detect a 1% seroprevalence with a 95% level of confidence at the level of the equid population using Epitools.^1^ This yielded animal sample sizes of 144 in Santa Cruz, 171 in Isabela, 79 in San Cristobal and 24 in Floreana. A mean number of three equids per farm was used to estimate the total number of farms to be included in the study. A minimum of 10 animals were sampled per farm and in farms with less than 10 animals, all animals were tested. The farms and the animals were randomly selected.

### Laboratory tests

Sera were tested using the commercial ID Screen West Nile competition multi-species ELISA kit (Innovative Diagnostics, Montpellier, France) according to manufacturer’s instructions. This method, that uses pre-coated plates with the envelop (E) protein of WNV, estimates the competition between antibodies present in the animal serum and monoclonal anti-WNV E antibody conjugated to horseradish peroxidase (HRP). This ELISA, through cross-reactions, allows the detection of other flaviviruses belonging to the Japanese encephalitis virus serocomplex as USUV (17). Assays were interpreted according to Beck et al. (18).

To detect antibodies against EIAV, serum samples were screened using a commercial ELISA, Equine Infectious Anemia Virus Antibody Test Kit, ELISA v2 (VMRD, Pullman, USA). Assays were performed according to the manufacturer’s instructions.

## Results

**Figure 1:**
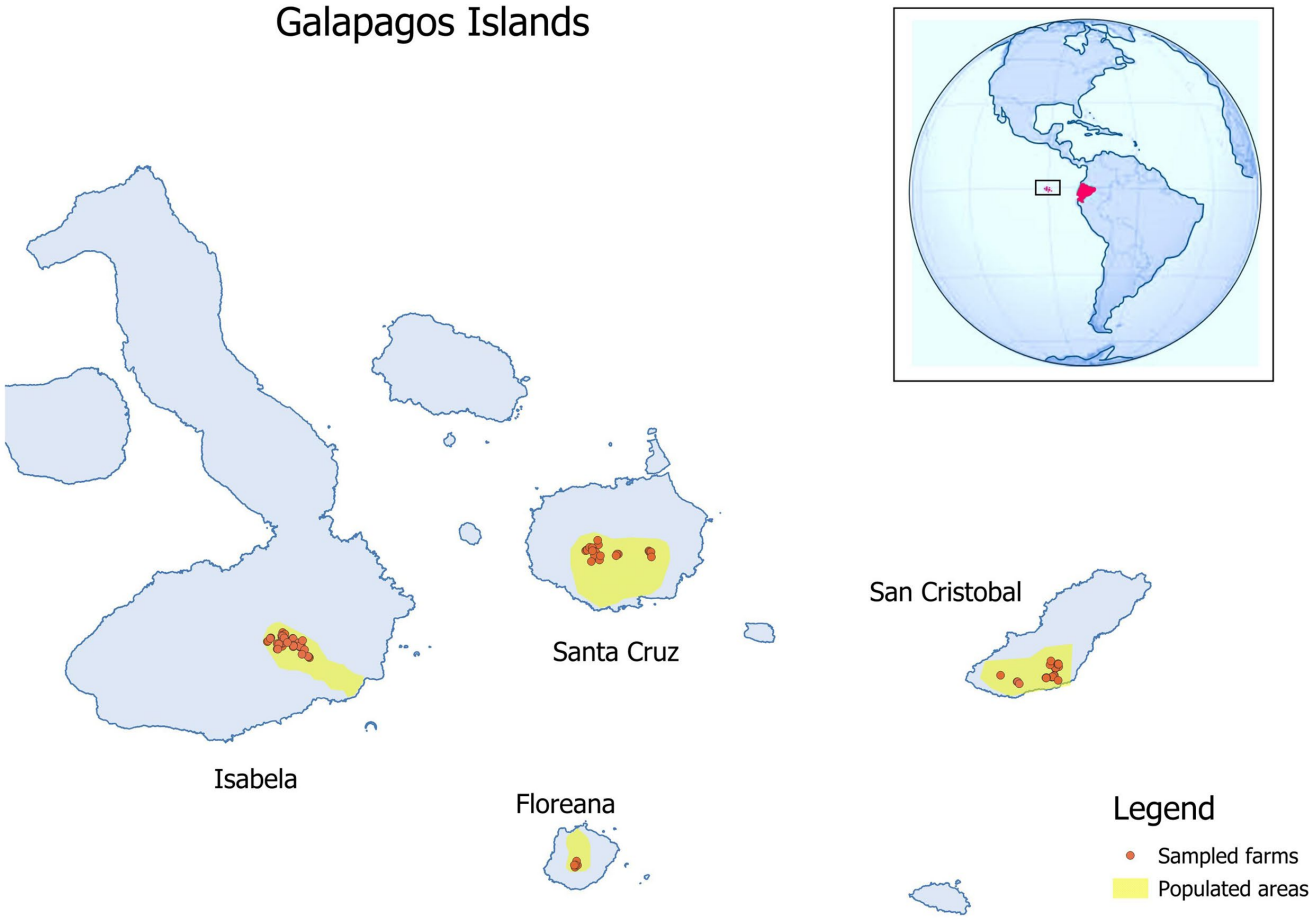
Location of the farms where equids where sampled to detect West Nile virus, Usutu virus and equine infectious anemia virus in 2019 in the Galapagos Islands. The georeferenced map was obtained in shape file format from the Geoportal of the Ecuadorian Military Geographic Institute and processed in Qgis 3.6.0 Noosa (QGIS.org, 2020).

Serum samples were collected from 411 equids (367 horses, 20 donkeys and 24 mules) in 124 farms in the four islands (Table 1; Figure 1) from April to July 2019. The sampled animals were between 3 months and 30 years old.

**Table 1:** Number of farms and equids sampled in the Galapagos Islands.

Island	No. of farms sampled	No. of equids sampled	Number of equids sampled per farm (min.–max.)	Age (min.–max.)
Santa Cruz	64	140	1–10	1 year–30 years
Isabela	25	171	1–36	3 months–30 years
San Cristóbal	29	80	1–10	3 months–20 years
Floreana	6	20	1–11	3 months–19 years
Total	124	411	1–36	3 months–30 years

All animals had negative results in the serological tests for the Flavivirus genus and EIAV.

## Discussion

The negative serological results obtained in this study suggest that the flavivirus that could affect equids, such WNV or USUV, and EIAV were not circulating in the Galapagos Islands above the prevalence value used to calculate the sample size for this study. This absence could go up to 30 years considering the age of some of the sampled equids.

The Galapagos Islands fill the climatic conditions favorable for the circulation of flaviviruses or EIAV provided the presence of competent vectors. The islands has three mosquito vectors capable of transmitting WNV and USUV (*Culex quinquefasciatus, Aedes aegypti, and Aedes taeniorhynchus*) (19, 20), bird species related to competent avian hosts (*Dendroica petechia*, *Mimus* spp) (21) and horseflies that can transmit EIAV (20). Since the climatic and vector conditions are filled, it could be argued that the prevalence viruses could be <1% in the equid population. However, in territories where those viruses are circulating, the prevalence values were higher than 1%. For example, WNV has been reported to be at seroprevalence values of 11% in horses in equids in Algeria (serums collected from 2015 to 2017) (22), 44.9% in Spain further to an outbreak in 2020 (23) or 31.6% in asymptomatic horses in Mexico (24). Analysis of 28,089 equids included in a systematic review and meta-analysis of seroprevalence studies of WNV in equids from 16 European countries between 2001 and 2018 revealed a pooled seroprevalence of 8% (25). In Morocco, prevalence of USUV was estimated to be 4% in a survey conducted in military working horses (26). In Spain, of 341 feral horses tested from 2010 to 2020, 9% were found seropositive to USUV (27). Regarding EIAV, in countries where this virus is circulating, prevalence values can be as high as 44% [survey conducted in Argentina (28)] but has also be found at lower values [2% in horses of the Para State in Brazil (14)]. On the other hand, according to the local veterinary services, there had been no reports of disease in equids in the Galapagos Islands consistent with WNV, USUV or EIAV or mortalities linked with WNV in birds.

The fact that the Galapagos Islands have semi-isolated conditions and the ban on animal importation do not provide sufficient guarantees to prevent the introduction of these viruses. Their introduction could arise by windborne transportation of infected vectors, infected migratory birds or infected vectors carried by airplanes or ships. These introduction routes were explored by Kilpatrick et al. (29) through a quantitative risk assessment to predict the WNV introduction into Galapagos Islands. These authors also included day-old chicks imports and mosquitoes in the larval stage present in shipment of tires as likely sources of WNV infection. They found that mosquitoes transported on airplanes carrying tourists represented the highest risk of WNV reaching the Galapagos Islands by a vector pathway and that the risk of introduction through migratory birds was lower but non-negligible. Moreover, it has been estimated that 107 Diptera species have arrived on the Galapagos Islands through human introductions and that 42 species have naturally colonized the islands from mainland Americas (20). Bataille et al. (30) showed from the monitoring of aeroplanes and genetic analysis that *C. quinquefasciatus* was regularly introduced via aircraft into the Galapagos islands. Therefore, effective control methods should be implemented by the national authorities to minimize the likelihood of introduction of insects through these routes that could put in danger endemic wildlife of Galapagos as well as domestic animal populations. Those measures could be combined with surveillance measures to detect any possible introduction. As the equid population is naïve to WNV and EIAV it is possible that that they could display clinical signs and, hence, equid owners should be made aware of the importance to report the occurrence of unexpected clinical cases. Future serum sampling carried out for other diseases in equids could also be used to detect flavivirus or EIAV. Hence, equids could act as sentinels to provide early warning of virus circulation.

## Data availability statement

The raw data supporting the conclusions of this article will be made available by the authors, without undue reservation.

## Ethics statement

The animal studies were approved by Agencia de Regulación y Control de la Bioseguridad y Cuarentena para Galápagos (ABG), Puerto Ayora, Ecuador. The studies were conducted in accordance with the local legislation and institutional requirements. Written informed consent was obtained from the owners for the participation of their animals in this study.

## Author contributions

GZ: Conceptualization, Data curation, Formal analysis, Investigation, Methodology, Validation, Writing – original draft, Writing – review & editing. CB: Formal analysis, Resources, Writing – review & editing. J-CV-C: Formal analysis, Resources, Writing – review & editing. MA: Formal analysis, Writing – review & editing. MC: Resources, Supervision, Validation, Writing – review & editing. AV: Resources, Supervision, Validation, Writing – review & editing. RV: Data curation, Validation, Writing – review & editing. GG: Formal analysis, Resources, Validation, Writing – review & editing.

## Footnotes

^1^ https://epitools.ausvet.com.au/
